# Abnormal expression of TGF-beta type II receptor isoforms contributes to acute myeloid leukemia

**DOI:** 10.18632/oncotarget.14325

**Published:** 2016-12-28

**Authors:** Yong Wu, Min Su, ShuX Zhang, Yu Cheng, Xiao Y. Liao, Bao Y. Lin, Yuan Z. Chen

**Affiliations:** ^1^ Fujian Institute of Hematology, Department of Hematology, Union Hospital, Fujian Medical University, Fuzhou, China

**Keywords:** TGF-β, type II receptor, isoform, myeloid, leukemia

## Abstract

Altered transforming growth factor-beta (TGF-β) signaling has been implicated in the pathogenesis of leukemia. Although TGF-β type II receptor (TβRII) isoforms have been isolated from human leukemia cells, their expression patterns and functions of these variants are unclear. In this study, we determined that two TβRII isoforms (TβRII and TβRII-B) are abnormally expressed in leukemic cells, as compared to normal hematopoietic cells. TβRII-B, but not TβRII, was found to promote cell cycle arrest, apoptosis, and differentiation of leukemic cells. TβRII-B also enhanced TGF-β1 binding and downstream signaling and reduced tumorigenicity *in vivo*. By contrast, TβRII blocked all-trans retinoic acid-induced differentiation through inhibition of TβRII-B. Overall survival was significantly lower in acute myeloid leukemia (AML) patients with high compared to low TβRII expression. Thus, whereas TβRII-B is a potent inducer of cell cycle arrest, apoptosis, and differentiation, higher TβRII expression correlates with poor clinical prognosis in AML.

## INTRODUCTION

Transforming growth factor-beta 1 (TGF-β1) is a potent inhibitor of hematopoietic cells. Autocrine- and paracrine-mediated binding of TGF-β1 to cell surface receptors regulates hematopoietic cell proliferation, differentiation, and apoptosis [1, 2]. TGF-β1 function is mediated by its interaction with transmembrane serine/threonine kinase receptors. It forms a heteromeric complex comprised of TGF-β receptor type I (TβRI) and type II (TβRII). TGF-β1 stimulation leads to phosphorylation of the transcription factors Smad2 and/or Smad3, which form a complex with Smad4. This complex translocates to the nucleus where it modulates the transcription of target genes by directly binding to DNA or interacting with promoter-specific transcription factors [3–5].

Altered TGF-β signaling has been observed in various human cancers [6–9]. However, the role of aberrant TGF-β signaling in leukemogenesis has not been investigated. The potential role(s) of TGF-β signaling in the pathogenesis of leukemia has attracted attention because TGF-β negatively regulates hematopoiesis [10, 11]. For example, in acute myeloid leukemia (AML), two distinct mutations in Smad4 (a missense mutation and a frameshift mutation) disrupt its ability to potentiate TGF-β transcriptional activity [12]. We previously reported that TGF-β1 expression was reduced in AML patients compared to healthy individuals. Interestingly, TGF-β1 expression returned to approximately normal levels in leukemia patients who achieved complete remission, but decreased after recurrence [13]. Other studies have demonstrated reduced TGF-β1 levels in several myeloid leukemia cell lines and in primary AML cells compared to normal cells. Ectopic expression of TGF-β1 in HL60 cells (human promyelocytic leukemia cells) inhibited proliferation and induced apoptosis both *in vitro* and *in vivo* [14]. Loss of TβRI and TβRII expression has also been observed in patients with myeloid leukemia [15]. Collectively, the data suggest that TGF-β signaling plays an important role in myeloid leukemogenesis.

An alternatively spliced variant of human TβRII (TβRII-B), which contains an insertion of 26 amino acids in place of Val32 of TβRII, was described previously [16]. Several studies have confirmed that TβRII-B is a functional TGF-β type II receptor that is expressed in a variety of cell lines [17–19]. We previously detected TβRII-B in human leukemia cells [20]. However, the expression patterns and functions of TβRII isoforms in leukemic cells have not yet been elucidated.

In this study, we examined the expression profiles of TβRII and TβRII-B in AML cells by real-time reverse transcription PCR (RT-PCR). Our data indicate that TβRII and TβRII-B are differentially expressed in AML and normal hematopoietic cells. TβRII-B is predominantly expressed in normal cells, while TβRII is primarily expressed in AML cells. We investigated the functions of the isoforms by stably expressing either TβRII or TβRII-B in K562 (myeloid leukemia) and HL60 (promyelocytic) cells. These cell lines were selected because they displayed low endogenous TβRII expression. We performed knock-down and rescue experiments in NB4 cells, which have high TβRII expression. These experiments revealed more pronounced TGF-β1-induced inhibition of proliferation and apoptosis in K562/TβRII-B and HL60/TβRII-B cells. Additionally, HL60/TβRII-B cells were more sensitive to all-trans retinoic acid (ATRA)-induced differentiation and As_2_O_3_-induced apoptosis. TβRII inhibited ATRA-induced differentiation of NB4 cells by blocking TβRII-B. Interestingly, TGF-β1 had a higher affinity for TβRII-B than TβRII, and HL60/TβRII-B cells exhibited reduced tumorigenicity *in vivo*. Thus, aberrant expression of TβRII isoforms in myeloid leukemia cells is associated with abnormal proliferation and differentiation, and inhibition of apoptosis. Additionally, high TβRII expression in leukemic cells is indicative of a poor prognosis in AML patients.

## RESULTS

### Analysis of the expression of TβRII and TβRII-B in primary myeloid leukemia cells, cell lines, and normal bone marrow CD34+ cells

**Figure 1:**
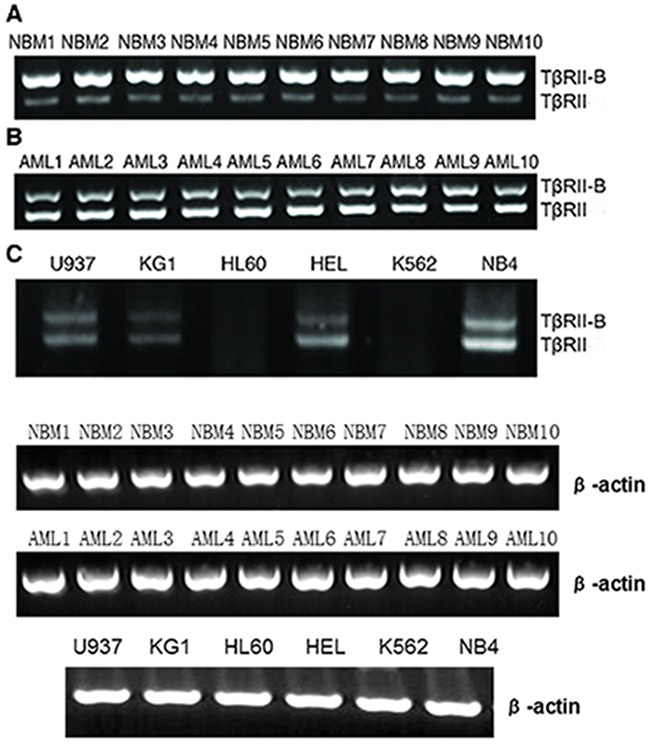
TβRII and TβRII-B expression in primary myeloid leukemia cells, cell lines, and normal bone marrow CD34+ cells TβRII and TβRII-B mRNA was detected in all primary AML cells, normal bone marrow CD34+ cells, and four AML cell lines, with the exception of K562 and HL60 cells (both TβRII isoforms were expressed at low levels). The leukemic cells and normal bone marrow cells differentially expressed TβRII. TβRII was mainly expressed in AML cells, whereas TβRII-B was mainly expressed in normal myeloid cells. **A**. NBM indicates normal bone marrow CD34+ cells. **B**. AML indicates bone marrow mononuclear cells with AML. **C**. TβRII was expressed at lower levels in K562 and HL60 cells than in other cell lines.

We first investigated the expression of TβRII isoforms in human leukemic cells using RT-PCR. Both TβRII and TβRII-B mRNA expression was detected in primary AML cells, normal bone marrow CD34+ cells, and four AML cell lines (U937, KG-1, NB4, and HEL). In contrast, both isoforms were expressed at low levels in K562 and HL60 cells. TβRII was differentially expressed in leukemic cells and normal bone marrow cells. TβRII was primarily expressed in AML cells while TβRII-B was predominantly expressed in normal myeloid cells (Figure 1A–1B). TβRII was expressed at lower levels in K562 and HL60 cells than in the other cell lines (NB4, U937, KG-1, and HEL) (Figure 1C). Based on these results, used K562 and HL60 cells for functional analyses of the two isoforms. We also performed knock-down and rescue experiments using NB4 cells, which have high TβRII expression, in order to investigate how the expression of TβRII impacted TβRII-B function.

### TβRII-B, but not TβRII, induces cell proliferation inhibition, apoptosis, and differentiation

**Figure 2:**
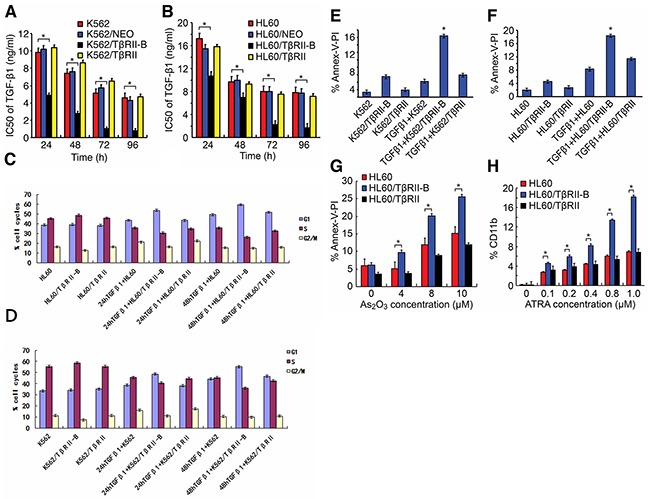
TβRII lacks the ability to induce cell proliferation inhibition, apoptosis, and differentiation **A**. and **B**. MTT assays were performed in triplicated and IC_50_ values for TGF-β1 calculated in K562 and HL60 cells that expressed either TβRII or TβRII-B. **C**. and **D**. Cell cycle analysis of K562 and HL60 cells expressing either TβRII or TβRII-B 24 h and 48 h after treatment with TGF-β1 (1 ng/mL). The flow cytometry data are expressed as percentages of the G1, S, and G2/M phases. The data are representative of three independent experiments. **E**. and **F**. FACS analysis of apoptosis using annexin V-FITC/PI staining in TGF-β1-treated (1 ng/mL) K562 and HL60 cells that stably expressed either TβRII or TβRII-B. The data are expressed as percentages of cells in early and late apoptosis. The data are representative of three independent experiments. **G**. Annexin V-FITC/PI staining for apoptosis induced by different concentrations of As_2_O_3_ in HL60 cells that expressed either TβRII or TβRII-B. The data are representative of three independent experiments. **H**. FACS analysis of ATRA-induced CD11b expression in HL60 cells that stably expressed the different TβRII isoforms. The FACS data are expressed as percentages of CD11b, and the data are representative of three independent experiments. The data are expressed as the mean ± SEM. **P < 0.01, *P < 0.05, two-tailed Student's *t*-test.

To elucidate the functions of the two isoforms, we stably expressed either TβRII or TβRII-B in the K562 and HL60 myeloid leukemia cell lines. Both cell lines have low endogenous TβRII expression. These cell lines (referred to herein as K562/TβRII, K562/TβRII-B, HL60/TβRII, and HL60/TβRII-B) were used to assess the impact on cell proliferation, apoptosis, and differentiation. TβRII and TβRII-B expression in these cells was confirmed using quantitative RT-PCR and Western blotting (Supplementary Tables 1 and 2, and Supplementary Figure 1A–1B). We performed MTT assays to evaluate the effects of TGF-β1 on the proliferation of K562 and HL60 cells that expressed either TβRII or TβRII-B. The cells were incubated with different concentrations of TGF-β1 for various times and the IC_50_ values calculated (Figure 2A–2B). Treatment with a low concentration of TGF-β1 (1 ng/mL) inhibited the proliferation of K562/TβRII-B and HL60/TβRII-B cells, whereas K562/TβRII and HL60/TβRII cells were not affected. We next performed cell cycle analysis (i.e. DNA ploidy) using flow cytometry (Figure 2C–2D, Supplementary Tables 3 and 4). After incubation with 1 ng/mL TGF-β1 for 24 h or 48 h, HL60/TβRII-B cells, but not HL60/TβRII cells, were arrested in the G1 phase. The same effect was observed in K562/TβRII-B and K562/TβRII cells. These data suggested that TβRII did not have the capacity to induce cell cycle arrest in AML cells.

To determine whether TGF-β1 could induce apoptosis in K562 and HL60 cells that expressed different TβRII isoforms, we measured apoptosis using annexin V-FITC/propidium iodide (PI) staining and flow cytometry (Figure 2E–2F). Apoptosis was observed in 16.49% and 18.26% of K562/TβRII-B and HL60/TβRII-B cells, respectively. Of the K562/TβRII and HL60/TβRII cells, 5.93% and 11.44%, respectively, were apoptotic. To study the effect of TβRII expression on As_2_O_3_-induced apoptosis, HL60 cells that expressed the different TβRII isoforms were incubated with various concentrations of As_2_O_3_ for 24 h. Cells that were double-stained with annexin V-FITC/PI were then detected using flow cytometry. A low concentration of As_2_O_3_ (4 μM) triggered apoptosis in HL60/TβRII-B cells but failed to induce apoptosis in HL60/TβRII and parental HL60 cells (Figure 2G). Apoptosis was also observed in HL60/TβRII and parental HL60 cells after treatment with higher As_2_O_3_ concentrations (8 μM and 10 μM)), but to a lesser extent than in HL60/TβRII-B cells. These data suggest that TβRII did not have the capacity to induce AML cell apoptosis.

**Figure 3:**
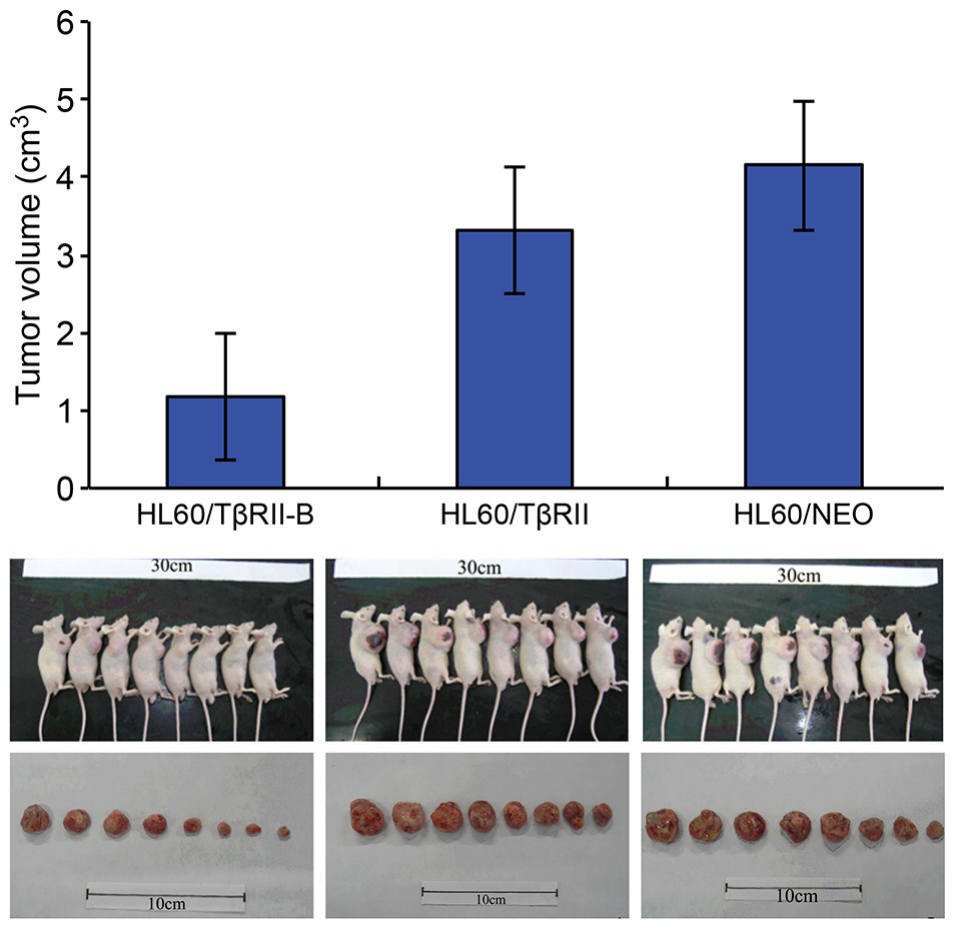
Growth of HL60/TβRII and HL60/TβRII-B cells in BALB/c nude mice 22 days post-inoculation The data are representative of two individual experiments. Each group contained eight mice. Representative images are shown. The data are expressed as the mean ± SEM. **P < 0.01, *P < 0.05, two-tailed Student's *t*-test.

We next investigated the response of HL60 cells that expressed different TβRII isoforms to ATRA-induced differentiation.HL60/TβRII and HL60/TβRII-B cells were first incubated with different concentrations of ATRA for 96 h. The expression of CD11b on the cell surface was then detected by flow cytometry. We found that 0.1–1.0 μM ATRA induced differentiation of HL60/TβRII-B cells but not HL60/TβRII or parental HL60 cells (Figure 2H). These data suggested that unlikeTβRII-B, TβRII lacked the capacity to inhibit proliferation, apoptosis, and differentiation *in vitro*. To investigate the ability of HL60 cells expressing different TβRII isoforms to proliferate *in vivo*, we inoculated the cells into nude mice and analyzed tumorigenesis. HL60/TβRII-B cells exhibited weak tumorigenicity compared to HL60/TβRII cells. Moreover, the tumor volumes in the HL60/TβRII-B group were significantly lower than in the HL60/TβRII and HL60/NEO group 22 days after inoculation (P < 0.05) (Figure 3).

### TβRII inhibits ATRA-induced differentiation of NB4 cells by blocking TβRII-B

To investigate how TβRII expression affected TβRII-B function, NB4 cells were stably infected with lentiviral vectors expressing TβRII siRNA and TβRII splice variants (Mu). The splice variants (Mu) contained synonymous mutations located in the sequences targeted by TβRII siRNA (Supplementary Figure 2). Therefore, TβRII siRNA could silence the expression of endogenous TβRII but not the expression of either TβRII splice variant (Mu). Coinfection of NB4 cells with lentiviral vectors expressing both TβRII splice variants (Mu) and TβRII siRNA resulted in stable expression of only the TβRII splice variants (Mu) and not endogenous TβRII. These cell lines are referred to herein as NB4/TβRII and NB4/TβRII-B.

**Figure 4:**
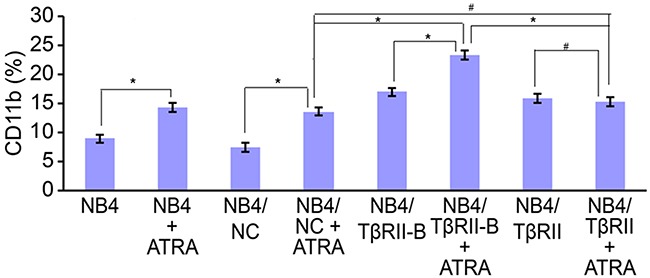
TβRII perturbs ATRA-induced differentiation of NB4 cells through blocking TβRII-B NB4 cells were infected with lentiviral vector for 96 h and then incubated with 0.1 μM ATRA for an additional 96 h. FACS analysis of ATRA-induced (0.1 μM) CD11b expression in NB4 cells after knock-down of endogenous TβRII and expression of the different TβRII isoforms. FACS data are expressed as percentages of CD11b expression, and the data are representative of three independent experiments. The data are expressed as the mean ± SEM. #P > 0.05, *P < 0.05, two-tailed Student's *t*-test.

NB4 cells were infected at a multiplicity of infection (MOI) of 20 for 96 h. The cells were then collected and the expression of TβRII splice variant mRNA analyzed in each group. We determined that both TβRII splice variants in the TβRII siRNA group were downregulated by 81% relative to cells infected with the negative control vector or the uninfected control cells. Real-time PCR analysis confirmed that the TβRII splice variants were stably expressed in the appropriate cell lines (Supplementary Figure 3). We next investigated the effects of ATRA on the differentiation of these lentiviral-infected NB4 cells. NB4 cells from each group were infected with lentiviral vectors for 96 h and then incubated with 0.1 μM ATRA for an additional 96 h. Finally, NB4 cell differentiation was analyzed by flow cytometry. We observed a higher number of differentiated cells in the NB4/TβRII-B group compared to the NB4/TβRII and negative control groups (P < 0.05). No ATRA-induced differentiation was observed in the NB4/TβRII group compared to the negative control group (P > 0.05) (Figure 4). These data showed that ATRA-induced differentiation of NB4/TβRII-B cells was enhanced by TβRII knock-down. Thus, TβRII perturbs ATRA-induced NB4 cell differentiation by inhibiting TβRII-B.

### TβRII lacks the ability to induce phosphorylation of TGF-β1/Smad pathway members and the expression of downstream target proteins

**Figure 5:**
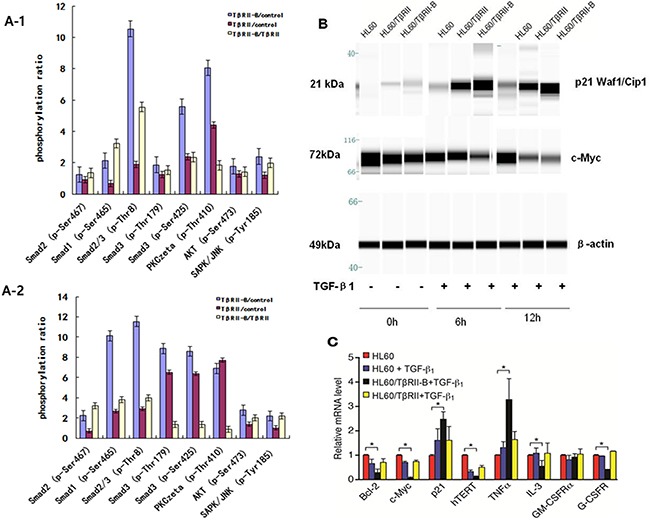
TβRII is deficient in inducing phosphorylation of TGF-β1/Smad pathway members and the expression of downstream target proteins **A1–2**. Phosphorylation of TGF-β1/Smad pathway members increased significantly in HL60/TβRII-B cells compared to HL60/TβRII cells after incubation with 1 ng/mL TGF-β1 for 0.5 h or 2 h. **B**. Western blot analysis of c-Myc and p21CIP/WAF1 expression in TGF-β1-treated (1 ng/mL) HL60 cells that stably expressed different TβRII isoforms. Untreated HL60 cells served as a control. The data are representative of two independent experiments. **C**. Real-time PCR analysis of TGF-β1 targets in HL60 cells expressing either TβRII or TβRII-B. Untreated HL60 cells served as a control. The experiment was performed in triplicate. The data are expressed as the mean ± SEM. *P < 0.05, two-tailed Student's *t*-test.

To study the molecular mechanisms underlying the functional differences in TGF-β signaling between the two different isoforms, we analyzed the effects of TGF-β1 on the phosphorylation of TGF-β1/Smad pathway members in HL60 cells that stably expressed the different TβRII isoforms. After incubation with 1 ng/mL TGF-β1 for 0.5 h or 2 h, phosphorylation of TGF-β/Smad signaling proteins was detected using an immunoassay consisting of 176 different antibodies (of which 74 antibodies targeted specific phosphorylation sites). The effects of TGF-β1 on the phosphorylation status of eight specific phosphorylation sites in six proteins known to be involved in TGF-β1/Smad signaling were investigated in HL60/TβRII or HL60/TβRII-B cells (Figure 5A and Supplementary Table 5). Phosphorylation of TGF-β1/Smad pathway members was significantly increased in HL60/TβRII-B cells compared to HL60/TβRII cells (Figure 1, 2). The phosphorylation status of TGF-β/Smad signaling proteins was confirmed by Western blotting (Supplementary Figure 4). Western blotting was also used to measure the levels of downstream targets of TGF-β signaling in HL60 cells that stably expressed the different TβRII isoforms. Treatment with 1 ng/mL TGF-β1 for 6 h or 12 h resulted in an increase in p21^CIP/WAF1^. To a greater extent in HL60/TβRII-B cells than in HL60/TβRII cells. In contrast, the levels of c-Myc levels significantly decreased, to a greater extent in HL60/TβRII-B cells than in HL60/TβRII cells. (Figure 5B).

To assess the effects of TGF-β1 on genes involved in cell cycle control, apoptosis, and differentiation, we measured the mRNA levels of Bcl-2, c-Myc, hTERT, p21, IL-3, TNFα, G-CSFR, and GM-CSFRα in HL60 cells that stably expressed the isoforms using quantitative RT-PCR (Figure 5C). Treatment of HL60/TβRII-B cells with TGF-β1 resulted in a decrease in Bcl-2, c-Myc, hTERT, IL-3, and G-CSFR mRNA. However, an increase in p21 and TNFα mRNA levels was observed. No significant changes were observed in HL60/TβRII cells.

### TGF-β1 has a higher affinity for TβRII-B than for TβRII

**Figure 6:**
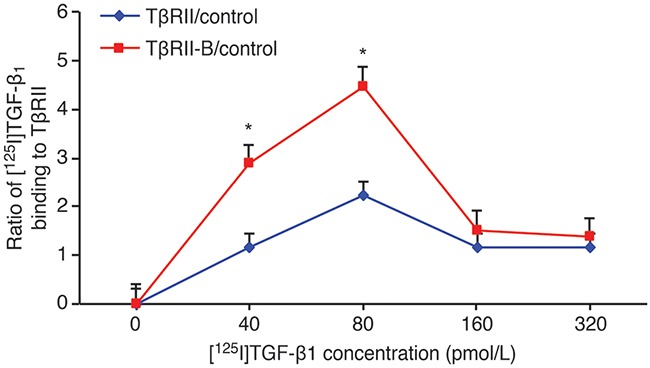
TGF-β1 has a higher affinity for TβRII-B than for TβRII Different concentrations of [^125^I]TGF-β1 were incubated with K562 cells that stably expressed TβRII or TβRII-B and the ratio of [^125^I]TGF-β1 binding calculated. K562 parental cells served as a control. The data are representative of two independent experiments. The data represent the mean ± SEM. *P < 0.05, two-tailed Student's *t*-test.

To study the binding affinity of TGF-β1 for TβRII and TβRII-B, different concentrations of [^125^I]TGF-β1 were incubated with K562/TβRII, K562/TβRII-B, or control K562 cells. These results indicated that 40–80 pmol/L (1–2 ng/mL) TGF-β1 bound more strongly to K562/TβRII-B cells than to K562/TβRII cells (Figure 6).

### Higher TβRII expression is correlated with a poor clinical prognosis in AML patients

**Figure 7:**
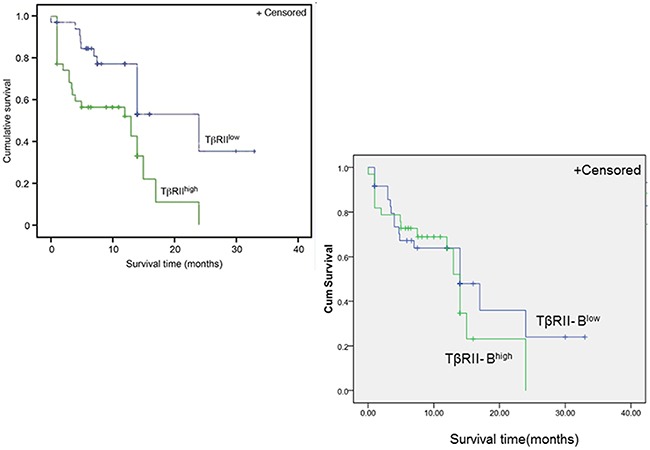
Higher TβRII expression is correlated with a poor clinical prognosis in AML patients Multivariate survival analysis of AML patients according to TβRII and TβRII-B expression. Kaplan-Meier survival curve. n = 138 patients. The overall survival rates of patients with high TβRII expression were significantly lower than those of patients with low TβRII expression (34.3% vs. 61.8%, P = 0.005). The overall survival rates of patients with high TβRII-B expression did not significantly differ from those of patients with low TβRII-B expression (45.5% vs. 50%, P > 0.05).

We next investigated whether the expression of TβRII isoforms was associated with AML patient prognosis. We analyzed TβRII and TβRII-B expression in 138 AML patients using quantitative RT-PCR. Of the 60 AML patients with high TβRII expression, 60% had high leukemic cell counts. Of the 78 patients with low TβRII expression, only 35.9% had high leukemic cell counts. Following treatment with two standard courses of chemotherapy, complete remission was observed in 40% of the AML patients who had high TβRII expression, which was a significantly smaller fraction than the fraction of patients with low TβRII expression who achieved remission (69.2%) (Table 1). Additionally, the overall survival rate of patients with high TβRII expression was significantly lower than that of patients with low TβRII expression (34.3% vs. 61.8%, P = 0.005) (Figure 7). These data indicated TβRII expression was significantly associated with patient prognosis. The characteristics of the AML patients (including genotype, gender, age, AML type, or karyotype) with high TβRII expression did not significantly differ from those of patients with low TβRII expression (Supplementary Table 6). In addition, the overall survival rates of patients with high TβRII-B expression did not significantly differ from those of patients with low TβRII-B expression (45.5% vs. 50%, P > 0.05). Finally, no significant differences in leukemic cell counts, complete remission, genotype, gender, age, AML type, or karyotype were observed between AML patients with high TβRII–B expression and patients with low TβRII-B expression.

**Table 1:** TβRII expression levels and clinical characteristics in AML patients

Patient characteristics	No. of patients (%)			P-value
	AML patients (n=138)	TβRII^low^ (n=78)	TβRII^high^ (n=60)	
Complete remission after two cycles of therapy				
Yes	78 (56.5%)	54 (69.2%)	24 (40%)	<0.01
No	60 (43.5%)	24(30.8%)	36 (60%)	
White blood cell count (×10^6^ cells /ml)				
Median	48.23	19.2	64.16	<0.05
Range	0.76-254.8	0.76-153.9	0.99-254.8	

TβRII^low^ and TβRII^high^ represent the 20% of patients with the lowest TβRII mRNA levels and the 20% with the greatest expression (the relative mRNA level is the ratio of the experimental data to the internal control data). P<0.01 and P< 0.05, *X^2^* analysis.

## DISCUSSION

Our data have revealed that TβRII and TβRII-B mRNA are abnormally expressed in AML cells and normal bone marrow CD34+ cells. TβRII was predominantly expressed in AML cells whereas TβRII-B was predominantly expressed in normal bone marrow CD34+ cells. Higher levels of TβRII and TβRII-B mRNA were also detected in U937, KG-1, HEL, and NB4 cells relative to K562 and HL60 cells. TβRII mRNA was also higher than TβRII-B in U937, KG-1, HEL, and NB4 cells. We transfected TβRII and TβRII-B splice variants into K562 and HL60 cells, which have relatively low TβRII expression, and generated the following cell lines: K562/TβRII, K562/TβRII-B, HL60/TβRII, and HL60/TβRII-B. Our data suggest that K562/TβRII-B and HL60/TβRII-B cells are more sensitive to TGF-β1-induced growth inhibition and apoptosis than K562/TβRII and HL60/TβRII cells.

We previously reported that ectopic expression of TGF-β1 in HL60, which lack endogenous TGF-β1 expression, inhibited cell proliferation and triggered apoptosis through downregulation of Bcl-2, c-Myc, and hTERT [14]. Here, we demonstrated that treatment with exogenous TGF-β1 downregulated Bcl-2, c-Myc, and hTERT mRNA expression to a greater extent in HL60/TβRII-B cells than in HL60/TβRII cells. As a cell cycle inhibitor, TGF-β1 not only suppresses the transcription of the *Bcl-2*, *c-Myc*, and *hTERT* genes, but also activates expression of the cell cycle inhibitor *p21^CIP/WAF1^*. We found that treatment with exogenous TGF-β1 reduced c-Myc protein expression and increased p21^CIP/WAF1^ protein levels to a greater extent in HL60/TβRII-B cells than in HL60/TβRII cells. TGF-β1 is known to regulate target gene expression through phosphorylation of R-Smad. We found that TGF-β1 led to increased R-Smad phosphorylation as well as phosphorylation of downstream targets including PKC, AKT, and SAPK/JNK in HL60/TβRII-B cells compared to HL60/TβRII cells.

Previous studies have suggested that TGF-β1 alone cannot induce significant HL60 cell differentiation. However, TGF-β1 in combination with low-dose ATRA (lower than therapeutic levels) could induce differentiation of HL60 cells [21]. We previously reported that ATRA-induced HL60 cell differentiation was associated with upregulation of endogenous TGF-β1, which suggested a synergistic relationship between the two reagents [22]. Combined TGF-β1 and vitamin D3 also had a stronger effect on differentiation in HL60 cells than either reagent alone. The combined effect on differentiation was primarily mediated by Smad2/3 phosphorylation and nuclear translocation [23]. Here, we found that HL60/TβRII-B cells were more sensitive to ATRA-induced differentiation than HL60/TβRII cells. These results suggested that ATRA-induced upregulation of endogenous TGF-β1 acted on TβRII signaling to induce cell differentiation. Other studies have suggested that Smad2/3 phosphorylation could be used as a sensor of TGF-β1- and ATRA-induced differentiation of HL60 cells into monocytes or myeloid cells. In our study, TGF-β1 alone induced differentiation of HL60 cells into monocytes. This effect was accompanied by rapid phosphorylation and nuclear translocation of Smad2/3. In contrast, ATRA induced the differentiation of HL60 cells into myeloid cells, but this process occurred without Smad2/3 phosphorylation and nuclear translocation. Treatment of HL60 cells with TGF-β1 plus ATRA resulted in differentiation of HL60 cells into both myeloid and monocyte cells [24]. The mRNA and protein levels of several TGF-β1 signaling pathway components including TGF-β1, TβRI, TβRII, Smad2, Smad4, and Smad7 were previously shown to increase in response to ATRA-induced NB4 cell differentiation. These results indicate that upregulation of TGF-β1 signaling pathway components plays an important role in ATRA-induced differentiation of NB4 cells.

We performed knock-down and rescue experiments in NB4 cells, which express high levels of endogenous TβRII, to investigate how TβRII expression impacted TβRII-B function. We determined that there was more ATRA-induced differentiation in NB4/TβRII-B cells compared to NB4/TβRII and negative control cells. In contrast, no effects were observed in NB4/TβRII cells compared to the negative controls. These data indicated that ATRA-induced differentiation of NB4/TβRII-B cells was enhanced by TβRII knock-down. Thus, TβRII perturbs ATRA-induced differentiation of NB4 cells through inhibition of TβRII-B.

We previously reported that As_2_O_3_ induced apoptosis in HL60 cells through upregulation of endogenous TGF-β1 and downregulation of Bcl-2. TGF-β1 antisense oligonucleotides inhibited As_2_O_3_-induced apoptosis and restored Bcl-2 mRNA and protein to pre-treatment levels, suggesting an important role for endogenous TGF-β1 in As_2_O_3_-induced HL60 cell apoptosis through downregulation of Bcl-2 [25]. Previous studies have also indicated that As_2_O_3_ and TGF-β1 exert a synergistic effect on apoptosis compared to either As_2_O_3_ or TGF-β1 treatment alone. Both As_2_O_3_ and TGF-β1 downregulate Bcl-2, suggesting that Bcl-2 is the common effector shared by the two apoptotic pathways [26]. TGF-β1 exerts this effect through its receptors. We found that HL60/TβRII-B cells were more sensitive to TGF-β1- or As_2_O_3_-induced apoptosis than HL60/TβRII cells.

In a physiological environment, hematopoietic stem cell proliferation, differentiation, and apoptosis are regulated by two types of cytokines: positive regulators (e.g. IL-1, IL-3, IL-6, G-CSF, GM-CSF, SCF, and TPO) and negative regulators (e.g. TGF-β1, TNFα, and IFNγ). These cytokines regulate blood cell proliferation, differentiation, and apoptosis in either an autocrine or paracrine manner. TGF-β1 downregulates the expression of the positive regulators to promote hematopoietic cell proliferation [27]. We previously found that TGF-β1 could decrease IL-3, GM-CSFR, IL-6, and IL-6R expression in leukemic cells [13], and we demonstrated that TGF-β1 increased TNFα mRNA expression and reduced IL-3 and G-CSFR mRNA expression in HL60/TβRII-B but not in HL60/TβRII cells. These findings suggest that leukemic cells expressing different TβRII isoforms may alter the effects of TGF-β1 on cytokines and cytokine receptors.

The structures of ternary complexes consisting of TGF-β type I and type II receptors and TGF-β1 have been solved. A comparison of the structure of the TGF-β1:TβRII:TβRI ternary complex with the TGF-β3 ternary complex revealed a common ligand recognition mode among receptors in the TGF-β cytokine family [28]. Groppe et al. [29] showed that TGF-β3 bound to the extracellular region of TβRI, and that the N-terminus of TβRII interacted with the C-terminus of TβRI to form a TGF-β3:TβRII:TβRI tertiary complex. Disrupting the interaction between these two receptors could block complex formation and downstream Smad signaling. The N-terminus of TβRII-B contains an insertion of 26 amino acids that replaces Val32, which corresponds to Val9 in the TβRII N-terminus. Here, binding assays indicated that at low TGF-β1 concentrations, the binding affinity of TGF-β1 for K562/TβRII-B cells was much stronger than for K562/TβRII cells. Thus, differences in binding of TβRII isoforms to TGF-β1 may affect the interaction between TβRII and TβRI and activation of downstream signaling pathways. To confirm that the two TβRII isoforms have different biological functions *in vivo*, we generated HL60 xenograft mouse models. We found that HL60/TβRII-B cells exhibited reduced tumorigenicity in nude mice compared to HL60/TβRII cells.

Because differences in the expression and function of the two TβRII isoforms have been documented in myeloid leukemia cells, we compared data on the clinical features and prognosis of 138 AML patients. AML patients with high TβRII expression had elevated white blood cell counts, lower complete remission rates, and lower overall survival rates than AML patients with low TβRII expression. Therefore, TβRII might be a prognostic marker in AML.

One limitation of this study is that the TβRII receptor subunits are overexpressed on myeloid leukemia cells. These cells do not normally express the receptor and therefore might not be physiologically primed to respond to TGF-β. Another potential limitation is that leukemic cells form solid tumors rather than hematopoietic cancers in the xenograft model. However, we simply used these cell lines and mouse models to elucidate the effects of the different isoforms on cell proliferation. Further studies involving intravenous transplantation of leukemia cells with varying TβRII expression should be performed to confirm our results. Additionally, the impact of targeting TβRII in leukemic cells could be investigated to analyze the potential therapeutic efficacy.

In conclusion, we have demonstrated that TβRII-B, but not TβRII, has the capacity to induce cell cycle arrest, apoptosis, and differentiation. Higher TβRII expression is correlated with a worse prognosis in AML patients.

## MATERIALS AND METHODS

### Patient population

A total of 138 previously untreated AML patients were selected at Union Hospital, which is affiliated with Fujian Medical University. The characteristics of the patients are shown in Supplementary Table 6. Bone marrow samples from 10 healthy donors were used as controls.

### Cell lines and separation of mononuclear cells

The K562 (ATCC No. CCL-243), HL60 (ATCC No. CCL-240), U937 (ATCC No. CRL-1593.2), KG1 (ATCC No. CCL-246), and HEL (ATCC No. TIB-180) cell lines were purchased from ATCC, and the NB4 cell line was purchased from the Institute of Cell Biology, Chinese Academy of Sciences. All cells were cultured in RPMI 1640 medium (Gibco/BRL) supplemented with 10% fetal bovine serum (FBS). Mononuclear cells were separated from 5 mL bone marrow samples using lymphocyte separation medium (specific gravity, 1.077 g/mL) according to the manufacturer's instructions.

### Isolation of CD34+ cells from normal bone marrow

Fluorescence activated cell sorting (FACS) was performed with a FACSAria I flow cytometer (Becton Dickinson) according to the manufacturer's protocol. CD34+ cells in the normal bone marrow cell fractions were sorted based on positive labeling with a FITC-anti-human CD34 antibody. The data for 10,000 events were then analyzed using the FlowJo software. The cells were sorted into 5 mL tubes containing 2 mL DMEM supplemented with 10% FBS, and the FSC threshold was set to exclude cell debris. The viability of the isolated cells was assessed using the trypan blue exclusion method. After sorting, the cells were collected, centrifuged, and resuspended in 10 mL cold DMEM/FBS. The tubes were stored in the dark on ice until RNA extraction was performed.

### RNA extraction, RT-PCR, and real-time PCR

Total RNA was extracted using TRIzol reagent (Invitrogen). The cDNA was synthesized using a reverse transcription kit (Toyobo). Isoform (TβRII and TβRII-B) expression was detected by RT-PCR. Specific primer sets were designed to detect different TβRII isoforms based on the presence of exon 2. The sequences of the primers are shown in Supplementary Table 2. The product length of TβRII-B was 234 bp and the product length of TβRII was 159 bp. Quantitative real-time PCR analysis of TβRII isoform (TβRII and TβRII-B) expression was performed using a model 7500 thermocycler (ABI). The primer and probe sequences are shown in Supplementary Table 7, and TaqMan probes (ABI) were used in all experiments. Different dilutions of the target genes as well as the internal control gene (*ABL1*) were included in order to calculate a standard curve. Quantitative analysis of mRNAs involved in cell cycle control, apoptosis, and differentiation was performed using a SYBR Green Real-time PCR kit (Toyobo). All experiments were performed according to the manufacturer's instructions. The primer sequences are shown in Supplementary Table 8. The relative transcript levels were calculated using the 2^-ΔΔCt^ method, with all data normalized to the level of β-actin.

### Generation of stable cell lines

K562 and HL60 cells were cultured in RPMI 1640 medium (Gibco/BRL) supplemented with 10% FBS. The pEFIRES-TβRII and pEFIRES-TβRII-B vectors were constructed by inserting the human TβRII and TβRII-B cDNA open reading frames, respectively, into a pEFIRES-Neo eukaryotic expression plasmid (gift from Ian G. Young, John Curtin School of Medical Research, Australian National University, Canberra, AU) and were confirmed by sequencing (BigDye Terminator 3.0, ABI). We transfected pEFIRES-TβRII and pEFIRES-TβRII-B into K562 and HL60 cells using the Lipofectin^TM^ reagent (Life Technologies). Cells were selected with hygromycin (100 μg/mL). Cell lines stably expressing TβRII or TβRII-B were screened using quantitative RT-PCR and Western blotting confirm TβRII expression.

### Lentiviral vector construction and infection

NB4 cells were cultured in RPMI 1640 medium (Gibco/BRL) supplemented with 10% FBS. Lentiviral vectors designed to express human TβRII siRNA or TβRII splice variants (Mu), and negative control vectors were constructed with the aid of Shanghai GeneChem. The siRNA sequences targeting TβRII were designed to silence both endogenous TβRII splice variants using an Invitrogen Neon Transfection System (MPK5000). A double-stranded oligonucleotide encoding the shRNA sequence, a mismatched siRNA mutant, and sequences encoding the two TβRII splice variants (Mu) tagged with green fluorescent protein (GFP) were annealed and inserted into the pGC-LV expression vector (Invitrogen). The siRNA and TβRII splice variant (Mu) expression vectors (pGC-LV), and the packaging vectors (pHelper 1.0 and pHelper 2.0; Invitrogen) were cotransfected into 293FT cells using Lipofectamine 2000 (Invitrogen). The culture supernatants were collected, concentrated, and stored at -70°C. Enhanced GFP (eGFP) was expressed in all lentiviral vectors to measure viral titer and infection efficiency. The lentiviral vectors were introduced into NB4 cells in the presence of polybrene (10 mg/mL), with an MOI of 5–20.

### Cell proliferation assays

MTT assays were performed using an MTT Cell Proliferation Assay Kit (Amresco) according to the manufacturer's instructions. Formazan absorbance was measured at 490 nm on an ELX800 microplate reader (BioTek). The following formula was used to quantify the effect on cell proliferation: cell proliferation inhibition rate = (1 - OD value of experimental group/OD of the control group) × 100%.

### Flow cytometry

Cell cycle analysis was performed using a cell cycle assay kit (Promega) according to the manufacturer's instructions. Flow cytometry cell cycle analysis was performed using a FACSAria I flow cytometer. The ModFit LT software was used to quantify DNA ploidy. Apoptosis was quantified using an Annexin V–FITC Apoptosis Detection Kit (Promega) according to the manufacturer's instructions. The data were analyzed using the FlowJo software (version 7.6.1; TreeStar). The cell surface differentiation antigen CD11b was detected by FACS. Briefly, 1 × 10^6^ cells were stained with FITC-anti-human CD11b for 20 min at room temperature. The cells were then washed and resuspended in FACS buffer. The cells were analyzed immediately after immunostaining using the FACSAria I and FlowJo Version 7.6.1 software.

### Western blotting

Total cell lysates were harvested and boiled in sample buffer. Equal amounts of protein lysate were resolved by SDS-PAGE and transferred onto polyvinylidene fluoride membranes (Millipore). The following primary antibodies were used in our analysis: anti-TβRII, anti-β-actin (Santa Cruz Biotechnology, Inc.), anti-p21CIP/WAF1, and anti-c-Myc (Cell Signaling Technology). Proteins were visualized by enhanced chemiluminescence (Amersham Biosciences).

### Phospho-protein profiling with a phospho-antibody array

Phospho-protein profiling was performed by the Biochip National Engineering Research Center of Shanghai, China. The TGF-β1/Smad Pathway Phosphorylation Antibody Array was designed and manufactured by Full Moon Biosystems, Inc. Total protein labeling, chip sealing, hybridization, and detection were performed according to the manufacturer's instructions. The microarrays were scanned with a GenePix 4000B array scanner and the mean values, CV values, and phosphorylation ratios calculated. The array contained 176 different antibodies, 74 of which targeted specific phosphorylation sites. For each antibody, we calculated the phosphorylation ratio using the following formula (phosphorylated and matching unphosphorylated values are denoted by “phospho” and “unphospho”, respectively, in both the control and the experimental data).

$$\text{phosphorylation ratio}=\frac{\text{phospho }(\text{experiment})}{\text{unphospho }(\text{experiment})}/\frac{\text{phospho}(\text{control})}{\text{unphospho }(\text{control})}$$

### [^125^I]TGF-β1 binding assays

The [^125^I]TGF-β1 was obtained from Beijing Atomic Hi-Tech Technology Co., Ltd., reconstituted in 100 mL 4 mM HCl containing 2 mg/mL BSA and diluted with binding buffer to generate a 100 pM stock solution. Aliquots were stored at -80°C until use. The [^125^I]TGF-β1 binding assays were performed as previously described [30]. To prepare cultures for binding studies, cells were seeded into six-well dishes and grown for 24 h. The medium was removed and the cells washed with 1 mL binding buffer and allowed to equilibrate at 4°C. After 30 min, the plates were transferred to ice and the binding buffer aspirated. Ice-cold binding buffer and [^125^I]TGF-β1 were added to different concentrations of [^125^I]TGF-β1 (range 5–320 pM). The final volume was 2 mL. The cells were then incubated at 4°C for 3.5 h on a platform that oscillated at 120 rpm. A 50-fold excess of unlabeled TGF-β1 was added immediately before the incubation with [^125^I]TGF-β1 in order to determine the amount of [^125^I]TGF-β1 that bound non-specifically to the surface of cells. In parallel, binding buffer without [^125^I]TGF-β1 was added to six-well plates for cell counting using a Neubauer counting chamber. Based on the work by Kang et al. [31], the following ratio of [^125^I]TGF-β1 binding to TβRII was calculated:

$$\text{TGF-ß1 binding ratio}=\frac{\text{binding }(\text{experiment})}{\text{binding }(\text{control})}$$

### *In vivo* assays of tumorigenesis

Female BALB/c nude mice (4–6 weeks old) were obtained from the Shanghai Laboratory Animal Breeding Center at the Chinese Academy of Medical Sciences. HL60/TβRII or HL60/TβRII-B cells (1 × 10^7^) were subcutaneously inoculated into the right flanks of the mice and tumor growth measured with a caliper. Tumors were allowed to grow until they became palpable (day 22). The mice were then sacrificed and the tumors removed and weighed. The tumor volume (cm^3^) was calculated according to Bhola et al.^32^ using the following formula: volume = length/2 × width^2^.

### Statistical analysis

All experiments were repeated with samples from at least two different cell preparations. All results are expressed as the mean ± standard error of the mean (SEM). Statistical significance was assessed using two-tailed Student's *t*-tests. The association between TβRII-B expression and overall survival was analyzed using the Kaplan-Meier method, and statistical significance was calculated based on log-rank tests. In all cases, P < 0.05 was considered significant.

### Study approval

All mice were maintained under pathogen-free conditions at Fujian Medical University, and all experiments were performed in accordance with the protocols of the Institutional Animal Care and Use Committee. Normal and clinical samples were collected from AML patients according to a protocol approved by the Fujian Medical University Ethics Committee. Written informed consent was also obtained.

## SUPPLEMENTARY TABLES AND FIGURES


