# Supplementary material for: Abnormal expression of TGF-beta type II receptor isoforms contributes to acute myeloid leukemia
Source: Oncotarget. 2016 Dec 28;8(6):10037–49. doi: 10.18632/oncotarget.14325 (PMC5354639; doi:10.18632/oncotarget.14325)
Supplement: Supplementary file 1 [file oncotarget-08-10037-s001.pdf]

Abnormal expression of TGF-beta type II receptor isoforms contributes to acute myeloid leukemia

SUPPLEMENTARY TABLES AND FIGURES

Supplementary Table 1: Quantitative RT-PCR analysis of TβRII mRNA in K562 cells stably expressing TβRII or TβRII-B

Cells	TβRII (CT value)	β-actin (CT value)	Fold change ($2^{-\Delta\Delta CT}$)
K562	30.98 ± 0.66	16.22 ± 0.43	1.00
K562/TβRII-B	22.13 ± 0.26	19.97 ± 0.56	6208.4 ± 540.76**
K562/NEO	31.73 ± 0.53	17.03 ± 0.24	1.04 ± 0.11
K562/TβRII	17.88 ± 0.33	16.04 ± 0.15	7750.10 ± 669.62**

The experiments were performed in triplicate. The data are expressed as the mean ± SEM. **P < 0.01, compared to K562 cells. K562/NEO is an empty vector control cell line.

Supplementary Table 2: Quantitative RT-PCR analysis of TβRII mRNA in HL60 cells stably expressing TβRII or TβRII-B

Cells	TβRII (CT value)	β-actin (CT value)	Fold change ($2^{-\Delta\Delta CT}$)
HL60	21.06 ± 0.15	15.47 ± 0.40	1.00
HL60/TβRII-B	16.65 ± 0.22	16.38 ± 0.28	39.95 ± 4.11**
HL60/NEO	20.39 ± 0.09	15.03 ± 0.38	1.17 ± 0.19
HL60/TβRII	15.52 ± 0.45	15.33 ± 0.33	42.22 ± 3.76**

The experiments were performed in triplicate. The data are expressed as the mean ± SEM. **P < 0.01, compared to HL60 cells. HL60/NEO is an empty vector control cell line.

Supplementary Table 3: Cell cycle analysis of HL60 cells stably expressing TβRII or TβRII-B after incubation with 1 ng/mL TGF-β1 for 24 h or 48 h

%	HL60	HL60/ TβRII- B	HL60/ TβRII	HL60 24 h	HL60/ TβRII-B 24 h	HL60/ TβRII 24 h	HL60 48 h	HL60/ TβRII-B 48 h	HL60/ TβRII 48 h
G1	38.57 ± 0.77	39.05 ± 0.16	38.12 ± 0.48	43.53 ± 1.02	53.55 ± 0.54	43.21 ± 1.29	49.16 ± 1.83	59.19 ± 0.76	51.62 ± 0.38
S	45.25 ±1.55	48.58 ± 0.25	45.78 ± 0.59	35.47 ± 0.74	30.41 ± 0.08	34.59 ± 0.44	35.52 ± 2.26	25.97 ± 0.86	32.61 ± 0.26
G2/M	16.18 ±1.75	12.37 ± 0.35	16.09 ± 0.26	21 ± 0.41	16.03 ± 0.50	22.21 ± 1.13	15.32 ± 0.46	14.84 ± 1.61	15.77 ± 0.63

The data are representative of two independent experiments.

Supplementary Table 4: Cell cycle analysis of K562 cells stably expressing TβRII or TβRII-B after incubation with 1 ng/mL TGF-β1 for 24 h or 48 h

%	K562	K562/ TβRII- B	K562/ TβRII	K562 24 h	K562/ TβRII-B 24 h	K562/ TβRII 24 h	K562 48 h	K562/ TβRII-B 48 h	K562/ TβRII 48 h
G1	33.57 ± 0.75	34.05 ± 0.15	35.12 ± 0.45	38.53 ± 1.08	48.55 ± 0.55	38.21 ± 1.30	44.16 ± 1.53	55.19 ± 0.96	46.62 ± 0.48
S	55.35 ± 1.35	58.68 ± 0.55	55.38 ± 0.69	45.57 ± 0.78	40.45 ± 0.28	44.49 ± 0.54	45.32 ± 1.26	35.87 ± 1.86	42.51 ± 1.26
G2/M	11.18 ± 1.05	7.37 ± 0.65	11.09 ± 0.56	16 ± 0.47	11.03 ± 0.55	17.21 ± 1.03	10.32 ± 0.86	9.84 ± 1.65	10.77 ± 0.68

The data are representative of two independent experiments.

Supplementary Table 5: Proteins that exhibited increased phosphorylation in HL60/TβRII-B cells compared to HL60/ TβRII cells

	p-Smad2 (Ser467)	p-Smad1 (Ser465)	p-Smad2/3 (Thr8)	p-Smad3 (Thr179)	p-Smad3 (Ser425)	p-PKCζ (Thr410)	p-AKT (Ser473)	p-SAPK/ JNK (Thr185)
TβRII-B / control 0.5 h	1.23	2.13	10.53	1.86	5.58	8.05	1.77	2.38
TβRII / control 0.5 h	0.91	0.66	1.90	1.22	2.38	4.39	1.26	1.2
TβRII-B / TβRII 0.5 h	1.35	3.22	5.54	1.52	2.34	1.83	1.41	1.98
TβRII-B / control 2 h	2.23	10.13	11.53	8.86	8.58	6.9	2.76	2.18
TβRII / control 2 h	0.71	2.66	2.90	6.52	6.38	7.7	1.36	1.0
TβRII-B/ TβRII 2 h	3.18	3.81	3.97	1.36	1.34	0.89	2.03	2.18

Supplementary Table 6: Clinical characteristics of AML patients and expression of TβRII expression

Patient characteristics	No. of patients (%)			P-value
	AML patients	TβRII^{low}	TβRII^{high}	
	(n = 138)	(n = 78)	(n = 60)	
Age, years				
Median	48	55	43	> 0.05
Range	17-79	19-79	17-77	
Sex				
Male	84 (60.9%)	48 (61.5%)	36 (60%)	> 0.05
Female	54 (39.1%)	30 (38.5%)	24 (40%)	
AML type				
APL	12 (8.7%)	10 (12.8%)	2 (3.3%)	> 0.05
No APL	126 (91.3%)	68 (87.2%)	58 (96.7%)	
M0	8 (5.8%)	4 (5.1%)	5 (8.6%)	
M1	16 (11.6%)	6 (7.7%)	10 (17.2%)	
M2	36 (26.1%)	20 (25.6%)	16 (27.6%)	
M4	4 (2.9%)	2 (2.6%)	3 (5.2%)	
M5	62 (44.9%)	36 (46.2%)	24 (41.3%)	
Karyotype				
Favorable + standard	128 (92.7%)	74 (94.9%)	54 (90%)	> 0.05
Unfavorable	10 (7.3%)	4 (5.1%)	6 (10%)	
FLT3 status				
FLT3 wild type	128 (92.7%)	74 (94.9%)	54 (90%)	> 0.05
FLT3 mutated	10 (7.3%)	4 (5.1%)	6 (10%)	
NPM-1 status				
NPM-1 wild type	132 (95.7%)	74 (94.9%)	58 (96.7%)	> 0.05
NPM-1 mutated	6 (4.3%)	4 (5.1%)	2 (3.3%)	

TβRII^{low} and TβRII^{high} represent the 20% of patients with the lowest and highest TβRII mRNA levels, respectively (the relative mRNA level is the ratio of the experimental data to the internal control data). $P < 0.01$ and $P < 0.05$, X^2 analysis.

Supplementary Table 7: Quantitative real-time PCR primers and probes

Primers	Sequence (5'–3')	Probe (5'–3')
TβRII-B	CGATCCCACCGCACGTT	CCACATCCGACTTCTG
	TCAGTGGATGGGCAGTCCTA	
TβRII	GCGTATCGCCAGCACGAT	TCAGAAGTCGGTTAATAAC
	CACCGTTGTTGTCAGTGACTATCA	
ABL1	GCCTACAACAAGTTCTCCATCAAGT	ACGTCTGGGCATTTGG
	CATAGGTAGCAATTTCCCAAAGCAA	

Supplementary Table 8: RT-PCR primers

Primers	Sequence (5'–3')	Length, bp
β-actin	GACTACCTCATGAAGATCCTCACC	85
	TCTCCTTAATGTCACGCACGATT	
TβRII	CACCGCACGTTCAGAAGTC	TβRII-B: 234
	CACAGATGGAGGTGATGCTG	TβRII: 159
Bcl-2	GGGTACGATAACCGGGAGAT	395
	CTGAAGAGCTCCTCCACCAC	
c-Myc	TCCTGGCAAAAGGTCAGAGT	265
	GTTGTGTGTTCGCCTCTTGA	
p21	ACCGAGACACCACTGGAG	203
	CGAGGCACAAGGGTACAA	
hTERT	CGGAAGAGTGTCTGGAGCAA	145
	GGATGAAGCGGAGTCTGGA	
TNFα	ATGGCGTGGAGCTGAGAGATA	382
	GGAAGGTTGGATGTTCGTCCT	
IL-3	CTTCAACAACCTCAATGGGG	441
	AATTCATCTGATGCCGCAGG	
GM-CSFRα	GCATTCCTCCTGATCCCAGAG	436
	CCCTCCTTCTCTTTGAGTTTCG	
G-CSFR	GGATCCGGGTCCATGGG	78
	TTAAGAGGCAGGCCCAAGAAG	

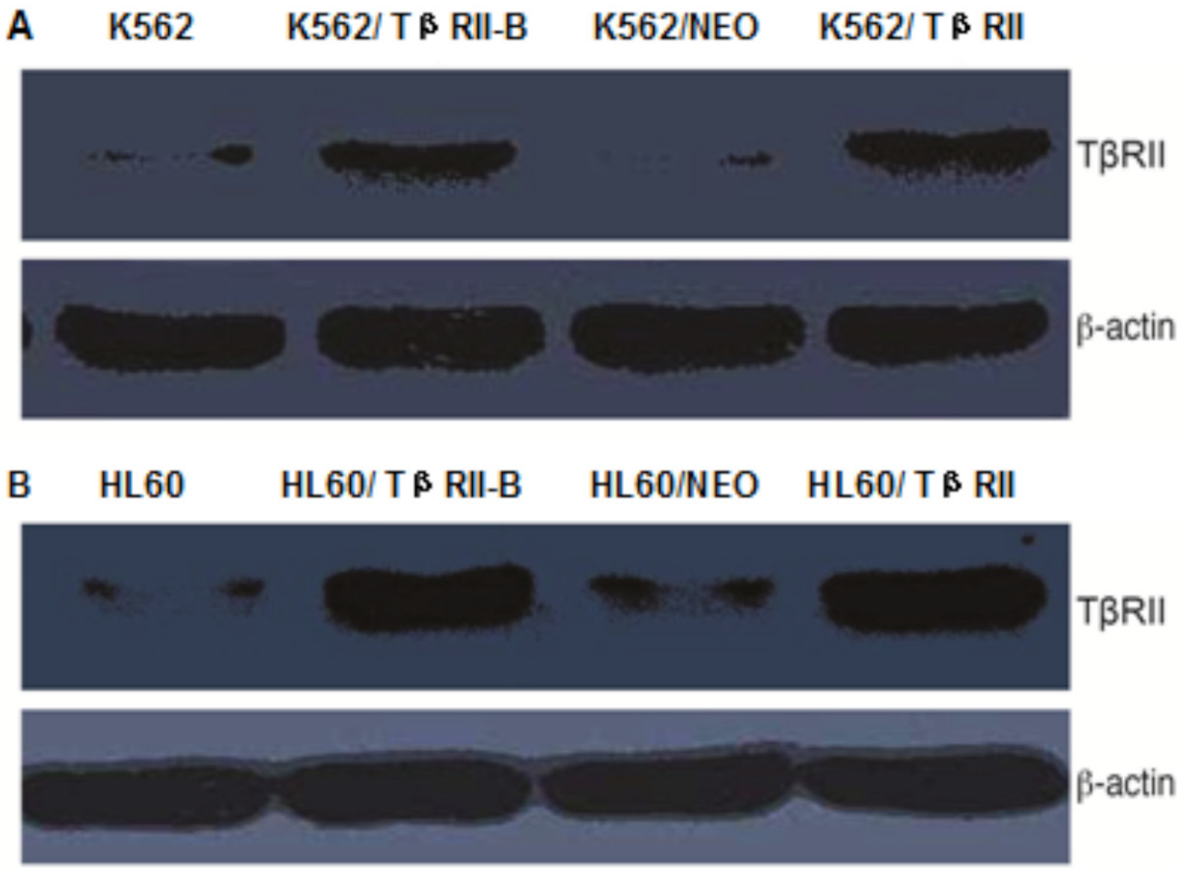

Supplementary Figure 1: Western blot analyses of TβRII expression in K562 and HL60 cells stably expressing TβRII or TβRII-B. A. TβRII expression in K562 cells stably expressing TβRII or TβRII-B. **B.** TβRII expression in HL60 cells stably expressing TβRII or TβRII-B. K562/NEO and HL60/NEO are empty vector control cell lines. The data are representative of two independent experiments.

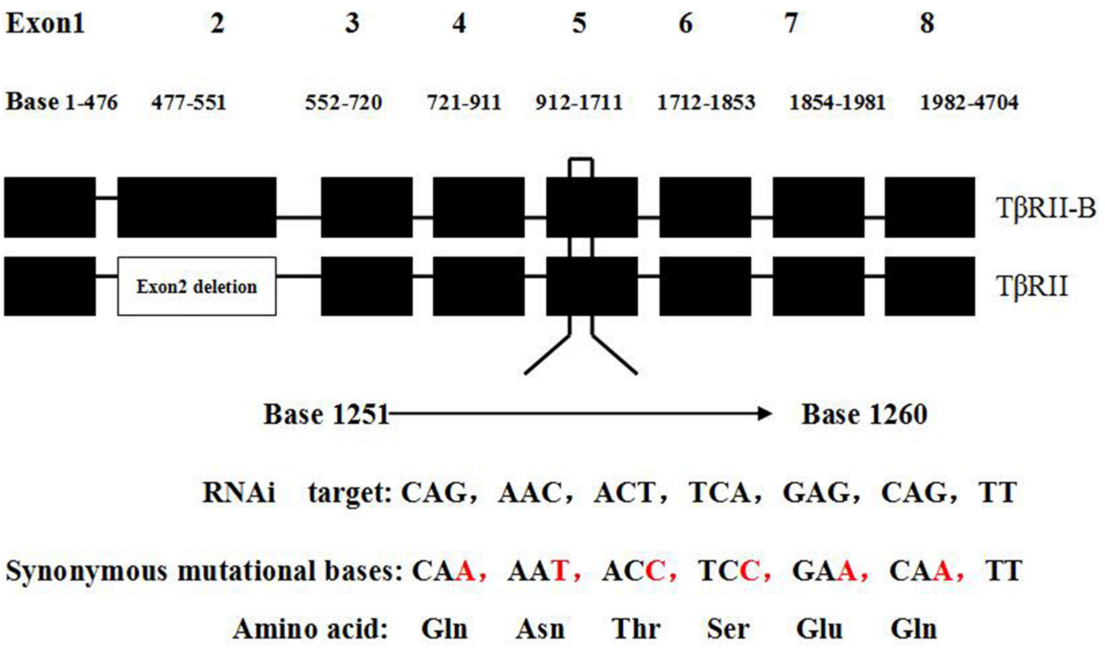

Supplementary Figure 2: Constructs for expression of TβRII splice variants containing synonymous mutations in the sequences targeted by TβRII siRNA.

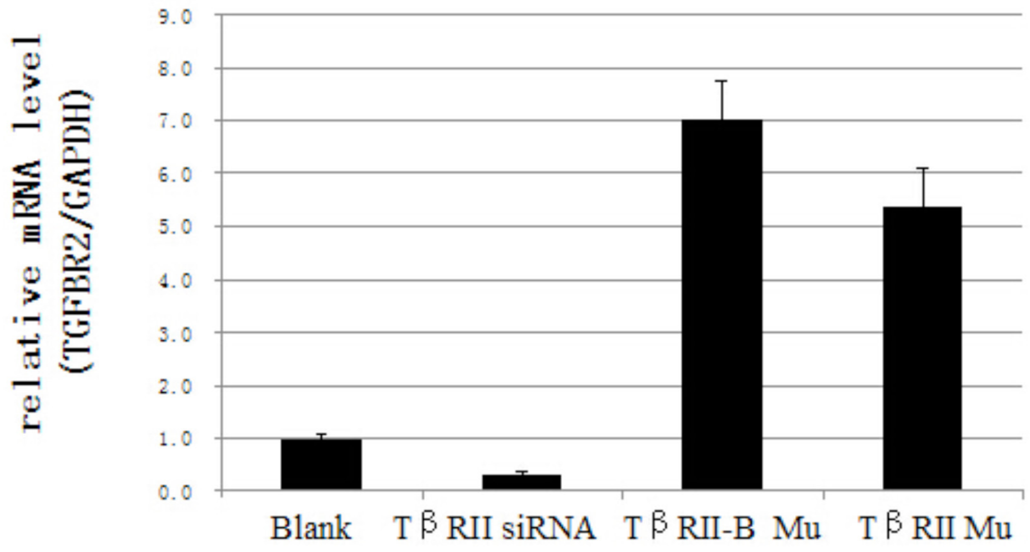

Supplementary Figure 3: Lentiviral infection of NB4 cells with TβRII siRNA and TβRII splice variants (Mu). At 96 hours post-infection, RNA was extracted and analyzed by RT-PCR. The relative expression of TβRII splice variants was quantified in each group by real-time PCR using isoform-specific primers.

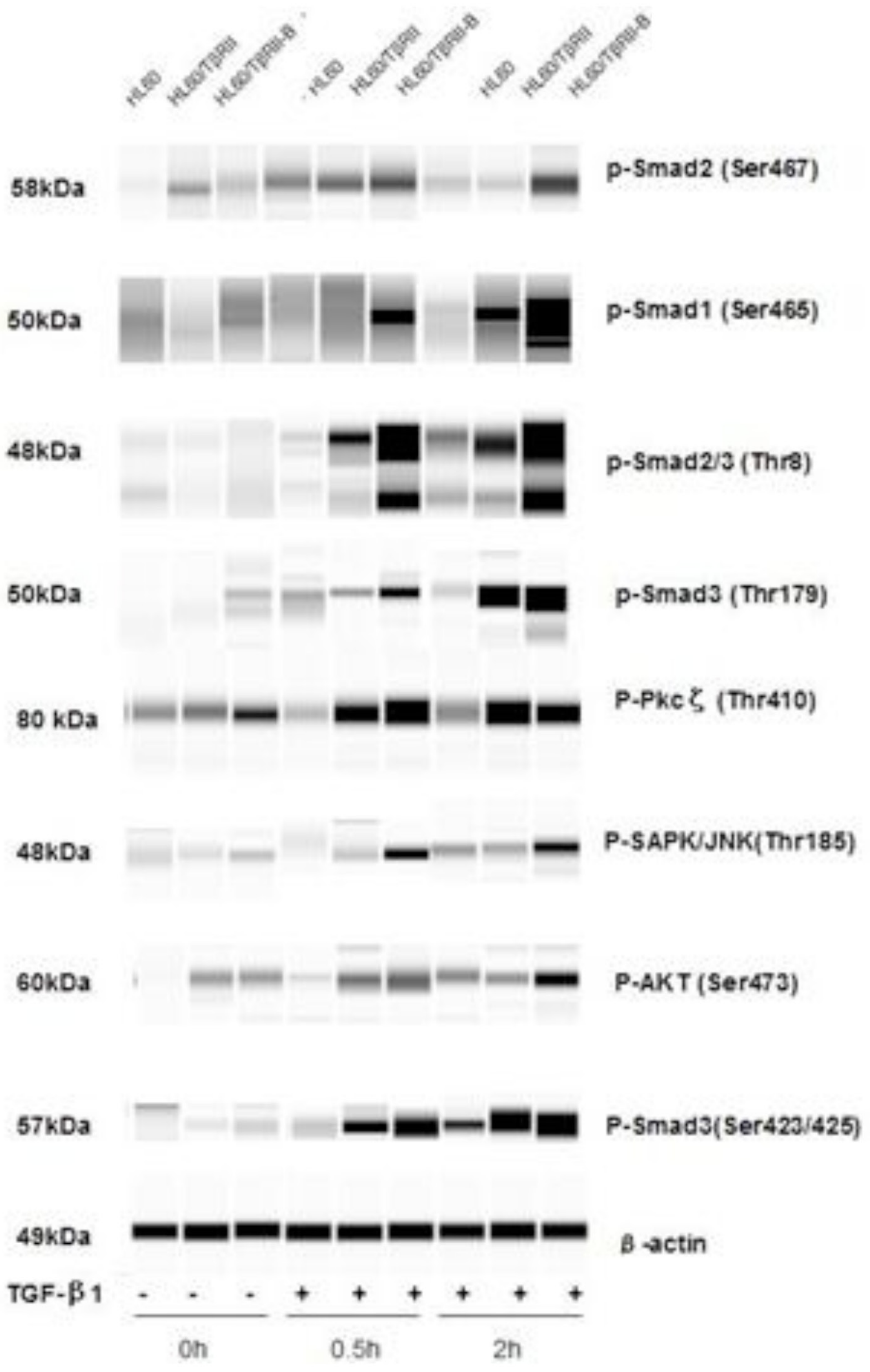

Supplementary Figure 4: Western blot analyses of protein phosphorylation in HL60/TβRII or HL60/TβRII-B cells after incubation with 1 ng/mL TGF-β1 for 0.5 h or 2h.
